# Atractylenolide III attenuates age-related vertebral bone loss by suppressing macrophage inflammation and osteoclastogenesis

**DOI:** 10.1016/j.bonr.2026.101955

**Published:** 2026-10-02

**Authors:** Cheng Xiang, Wenyuan Xiang, Jannatul Ferdus Shila, Atikul Islam, Yilin Liao, Zhengxiao Ouyang, Gen Wu, Xinfeng Gao

**Affiliations:** a Department of Orthopedics, Wuhan Fourth Hospital, Wuhan, 430033, Hubei, China; b Hubei Provincial Clinical Research Center for Orthopaedics, China; c Department of Orthopedics, Xinjiang Medical University Affiliated Traditional Chinese Medicine Hospital, Urumqi, 830000, Xinjiang, China; d Department of Microbiology, Rabindra Maitree University, Kushtia, 7000, Bangladesh; e Department of Pathology and Laboratory Medicine, University of Rochester Medical Center, Rochester, NY, 14642, USA; f Department of Orthopedics, The Second Xiangya Hospital, Central South University, Changsha, 410011, Hunan, China

**Keywords:** Atractylenolide III, Skeletal aging, Bone marrow macrophages, Osteoclastogenesis, Vertebral microarchitecture, TLR4/NF-κB

## Abstract

Age-related bone loss is driven in part by inflammatory remodeling of the bone marrow niche, which promotes macrophage activation and osteoclastogenesis. Atractylenolide III (AT-III) is a chemically defined sesquiterpene lactone with anti-inflammatory activity, but its skeletal effects in natural aging remain unclear. Bone marrow cells and macrophages from young and aged mice were evaluated by flow cytometry, ELISA, immunostaining, TRAP staining, RT-qPCR, and immunoblotting. Twenty-four-month-old male mice received vehicle or AT-III (10 or 20 mg/kg/day) for 1 month, followed by vertebral micro-computed tomography and histomorphometry. Aged bone marrow exhibited increased TLR4 and IL-1β signals in CD11b-positive myeloid cells, particularly a macrophage-enriched population. In aged bone marrow-derived macrophages, AT-III reduced TNF-α and IL-1β secretion, lowered TLR4 and IL-1β staining, and suppressed RANKL-induced osteoclast formation. AT-III-treated aged mice showed improved vertebral microarchitecture, increased osteoblast surface, and reduced osteoclast and eroded-surface indices without overt short-term changes in body weight or major-organ histology. AT-III also reduced phosphorylation of RelA and p50 and decreased Tlr4, Rela, and Nfkb1 expression. Exploratory network pharmacology and molecular docking independently prioritized TLR4/NF-κB-related signaling. These findings indicate that AT-III attenuates age-related vertebral bone loss and inflammatory osteoclastogenesis, while direct target engagement and causal pathway dependence remain to be established.

## Highlights

• AT-III protects vertebral microarchitecture in naturally aged male mice.
• AT-III suppresses inflammatory signaling in aged bone marrow macrophages.
• AT-III reduces RANKL-induced osteoclastogenesis in aged marrow cultures.
• TLR4/NF-κB suppression is supported but not established as causal.

## 1 Introduction

Age-related bone loss reflects progressive uncoupling of skeletal remodeling, with excessive bone resorption and an inadequate formation response. Inflammaging and age-associated remodeling of the marrow niche promote myeloid bias and disturb communication between immune and skeletal cells (Franceschi et al., 2018; Ambrosi et al., 2021; Miron et al., 2024). Macrophages are central to this process because they coordinate inflammatory responses and provide osteoclast precursors. Sustained macrophage activation can therefore enhance osteoclast formation and accelerate deterioration of bone microarchitecture.

Atractylenolide III (AT-III) is a purified, chemically defined sesquiterpene lactone isolated from Atractylodes species (Zhang et al., 2021b; Deng et al., 2021). Experimental studies have described anti-inflammatory and immunomodulatory effects of AT-III in pulmonary, intestinal, esophageal, and osteoarthritis-related models (Huai and Ding, 2020; Dai et al., 2025; Si et al., 2023; Xu et al., 2023). These findings suggest that AT-III may regulate inflammatory pathways relevant to skeletal aging; however, its effects on aged bone marrow macrophages and age-related bone loss have not been established.

Toll-like receptor 4 (TLR4)-dependent nuclear factor-κB (NF-κB) signaling is a major inflammatory pathway in macrophages. Persistent pathway activation can increase production of interleukin-1β (IL-1β), tumor necrosis factor-α (TNF-α), and other mediators that favor osteoclastogenesis. Network pharmacology can help prioritize compound-target-pathway relationships, but computational predictions do not establish direct binding or pathway necessity and must be interpreted as hypothesis-generating evidence (Hopkins, 2008; Nogales et al., 2022).

In this study, we investigated whether AT-III attenuates age-related bone loss by suppressing macrophage-associated inflammation and osteoclastogenesis. We combined experiments in naturally aged mice and aged bone marrow-derived macrophages with exploratory target prediction and molecular docking. Particular attention was given to TLR4/NF-κB-related signaling, while causal claims were limited to the level supported by the available pharmacological and molecular data.

## 2 Materials and methods

### 2.1 Reagents and antibodies

Atractylenolide III (AT-III; catalog no. HY-N0203; MedChemExpress) was dissolved in dimethyl sulfoxide (DMSO) to prepare a 100-mM stock solution and stored at −20 °C. α-Minimum Essential Medium (α-MEM), fetal bovine serum (FBS), penicillin-streptomycin, phosphate-buffered saline (PBS), and 0.25% trypsin were obtained from Gibco. The Cell Counting Kit-8 (CCK-8) was purchased from MedChemExpress. Antibodies against RelA/p65 (AF5006), phospho-RelA/p65 (3033 T), p50 (AF6217), phospho-p50 (AF3219), and GAPDH (60004–1-Ig) were obtained from Affinity Biosciences, Cell Signaling Technology, or Proteintech, as applicable. Antibodies used for flow cytometry and immunostaining are described below.

### 2.2 Animals and experimental design

Male C57BL/6 mice aged 24 months were obtained from the Laboratory Animal Center of Xinjiang Medical University (production license no. SCXK[Xin]2023–0002). Fifteen aged mice (28–35 g) were randomly allocated to vehicle, low-dose AT-III, or high-dose AT-III groups (*n* = 5 per group). Vehicle or AT-III (10 or 20 mg/kg/day) was administered by intraperitoneal injection for 1 month. Body weight was recorded weekly. At the end of treatment, mice were euthanized by gradual CO₂ inhalation followed by cervical dislocation. The heart, liver, spleen, lung, and kidneys were collected for hematoxylin and eosin (H&E) staining as a preliminary assessment of overt short-term visceral toxicity. The first lumbar vertebra (L1) was selected as the primary skeletal site and collected for micro-computed tomography (μCT) and histomorphometry.

### 2.3 Micro-computed tomography and bone histomorphometry

L1 vertebrae were fixed in 10% paraformaldehyde for 48 h and transferred to 70% ethanol before high-resolution μCT scanning (Scanco μCT40; 10-μm voxel size, 50 kV, 500 μA). Three-dimensional reconstruction and morphometric analysis were used to quantify bone volume fraction (BV/TV, %), trabecular number (Tb.N, mm − 1), trabecular thickness (Tb.Th, μm), trabecular separation (Tb.Sp, μm), and cortical thickness. After μCT, vertebrae were decalcified in 14% EDTA at 4 °C for 3 weeks, dehydrated, paraffin embedded, and sectioned at 4 μm. Osteoblast-related morphology was evaluated by H&E staining, and osteoclasts were assessed by tartrate-resistant acid phosphatase (TRAP) staining. Osteoblast surface (Ob.S/BS, %), osteoclast surface (Oc.S/BS, %), osteoclast number (Oc.N/BS, N/mm), and eroded surface (ES/BS, %) were quantified using OsteoMeasure software (OsteoMetrics).

### 2.4 Flow cytometry

Bone marrow cells were isolated from young (3-month-old) and aged (24-month-old) mice (*n* = 3 per age group). Bone marrow was flushed with cold PBS, passed through a 70-μm cell strainer, and centrifuged at 500 ×*g* for 5 min at 4 °C. Red blood cells were lysed with ammonium chloride buffer for 5 min at room temperature. After washing in PBS containing 2% FBS, 1 × 10^6^ cells were stained with fluorochrome-conjugated antibodies against Ly6G-APC-Cy7, F4/80-BV605, CD11b-PerCP-Cy5.5, IL-1β-PE, and TLR4-FITC (BioLegend). Samples were analyzed on a BD LSRFortessa flow cytometer, and data were processed using FlowJo version 10.9.0. The CD11b^+^Ly6G^−^F4/80^+^ population was operationally defined as macrophage enriched, whereas CD11b^+^Ly6G^+^F4/80^−^ cells were considered neutrophil enriched.

### 2.5 Isolation of aged bone marrow-derived macrophages and cell viability

Bone marrow cells from aged mice were cultured in α-MEM containing 10% FBS, 1% penicillin-streptomycin, and 20 ng/mL macrophage colony-stimulating factor (M-CSF) at 37 °C in 5% CO₂. Medium was partially replaced every 3 days. After 7 days, adherent bone marrow-derived macrophages (BMMs) were harvested, counted, and seeded into 96-well plates at 5 × 10^3^ cells per well. After overnight attachment, cells were treated for 24 h with serial twofold dilutions of AT-III starting at 200 μM. Viability was assessed by CCK-8 absorbance at 450 nm. Based on these data, 25 and 50 μM AT-III were selected for subsequent experiments.

### 2.6 Cytokine measurement by ELISA

After 24 h of treatment with AT-III (25 or 50 μM), culture supernatants were collected and centrifuged at 1000 ×*g* for 5 min at 4 °C. TNF-α (catalog no. 88–7324-88) and IL-1β (catalog no. 88-7013 A-88) were quantified using mouse ELISA kits (Invitrogen) according to the manufacturer's instructions. Standards and samples were analyzed in duplicate, absorbance was read at 450 nm with a 570-nm reference, and cytokine concentrations were derived from standard curves.

### 2.7 Immunofluorescence and immunohistochemistry

Bone marrow cells were seeded on chamber slides and treated with AT-III (25 or 50 μM) for 24 h. Cells were fixed, permeabilized, blocked, and incubated overnight at 4 °C with antibodies against TLR4 (MA5–16216) and IL-1β (P420B). Fluorophore-conjugated secondary antibodies were applied, and nuclei were counterstained with DAPI. Images were acquired under identical exposure conditions. Vertebral sections from vehicle- and AT-III-treated mice were processed for immunohistochemical detection of TLR4 and IL-1β, followed by horseradish peroxidase-conjugated secondary antibodies, DAB development, and hematoxylin counterstaining. Staining was quantified in ImageJ using color deconvolution, a uniform threshold, and integrated optical density; three randomly selected fields per sample were analyzed in a blinded manner.

### 2.8 In vitro osteoclastogenesis

Bone marrow cells were cultured in α-MEM containing 10% FBS, 1% penicillin-streptomycin, 1% non-essential amino acids, 1% l-glutamine, and 20 ng/mL M-CSF. After 2 days, macrophages were induced with 10 ng/mL receptor activator of NF-κB ligand (RANKL) for 5 days in the presence or absence of AT-III. Cells were fixed and stained using a TRAP kit. TRAP-positive multinucleated cells containing at least three nuclei were counted as osteoclasts, and osteoclast number and total TRAP-positive area were quantified using ImageJ.

### 2.9 In vitro osteogenic differentiation assay

To evaluate the potential direct effect of AT-III on osteogenic differentiation, BMSCs isolated from aged (24-month-old) mice were cultured in osteogenic induction medium supplemented with 50 μg/mL vitamin C (Sigma-Aldrich, A4403) and 10 mM β-glycerophosphate (β-GP, Sigma-Aldrich, G9422) and treated with vehicle, 25 μM AT-III, or 50 μM AT-III for 5 days. At the endpoint, ALP staining was performed and the ALP-positive area was quantified with ImageJ as a percentage of the total culture area. For immunofluorescence, cells were stained for COL1 (Invitrogen, MA1–26771) and RUNX2 (Invitrogen, MA5–41185), counterstained with DAPI, and imaged under identical exposure settings. Relative fluorescence intensity was quantified using ImageJ. For gene-expression analysis, total RNA was isolated, reverse transcribed, and analyzed by RT-qPCR. Col1 and Runx2 expression levels were normalized to GAPDH using the 2^-ΔΔCt^ method.

### 2.10 Immunoblotting and RT-qPCR

BMMs were treated overnight with AT-III and lysed in RIPA buffer containing protease and phosphatase inhibitors. Protein concentrations were measured by BCA assay. Equal amounts of protein (40 μg) were separated by 10% SDS-PAGE, transferred to PVDF membranes, blocked in 5% non-fat milk, and incubated overnight at 4 °C with antibodies against total and phosphorylated RelA and p50, with GAPDH as the loading control. Signals were visualized by enhanced chemiluminescence and quantified with Image Lab software. For RT-qPCR, total RNA was isolated using TRIzol, reverse transcribed, and analyzed with SYBR Green chemistry. Tlr4, Rela, and Nfkb1 expression was normalized to GAPDH and calculated by the 2^−ΔΔCt^ method. All primer sequences are provided in Supplementary Table 2.

### 2.11 Exploratory network pharmacology and molecular docking

Computational analyses were performed to generate mechanistic hypotheses rather than to establish direct target engagement. Potential AT-III targets were predicted using PharmMapper from the chemical structure, with z-score > 0 used as the screening threshold (Liu et al., 2010; Bahuguna et al., 2020). Genes associated with age-related osteoporosis were retrieved from GeneCards (Stelzer et al., 2016), and overlapping targets were identified using Venny 2.1.0. A protein-protein interaction network was generated in STRING and visualized in Cytoscape; clusters were explored using MCODE (Shannon et al., 2003). Gene Ontology and Kyoto Encyclopedia of Genes and Genomes enrichment analyses were performed in Metascape with Benjamini-Hochberg false-discovery-rate correction (q < 0.05), and selected pathway relationships were visualized using KEGG Mapper (Kanehisa and Sato, 2020). Molecular docking of AT-III with selected candidate proteins was performed using SwissDock, with protein structures from the Protein Data Bank and the ligand structure from PubChem. Docking ranks and representative poses were interpreted only as supportive computational evidence.

### 2.12 Statistical analysis

Data are presented as mean ± standard deviation unless otherwise indicated. In vitro experiments were performed in at least three independent biological replicates unless stated otherwise. Comparisons between two groups were performed using an unpaired two-tailed Student's *t*-test. Comparisons among three or more groups were performed using one-way analysis of variance followed by Bonferroni-adjusted post hoc comparisons. A two-sided *P* value <0.05 was considered statistically significant.

### 2.13 Ethics approval

All animal procedures were approved by the Institutional Animal Care and Use Committee of Xinjiang Medical University (approval no. IACUC-20240227-58) and were conducted in accordance with institutional animal welfare requirements and the ARRIVE guidelines.

## 3 Results

### 3.1 Aging increases TLR4 and IL-1β signals in macrophage-enriched bone marrow myeloid cells

We first examined whether the aged bone marrow microenvironment showed evidence of macrophage-associated inflammatory activation. The gating strategy and single-stain thresholds are shown in Fig. 1A. TLR4 and IL-1β signals were concentrated predominantly in CD11b-positive myeloid cells rather than CD11b-negative cells and were higher in aged than in young bone marrow (Fig. 1B, D). Subdivision of the CD11b-positive compartment using Ly6G and F4/80 identified neutrophil-enriched and macrophage-enriched populations (Fig. 1C). The CD11b^+^Ly6G^−^F4/80^+^ macrophage-enriched population exhibited greater TLR4 and IL-1β signals than the CD11b^+^Ly6G^+^F4/80^−^ neutrophil-enriched population, with an age-associated increase (Fig. 1E-I). These findings support macrophage-enriched myeloid cells as an important component of inflammatory signaling in aged bone marrow.

**Fig. 1:**
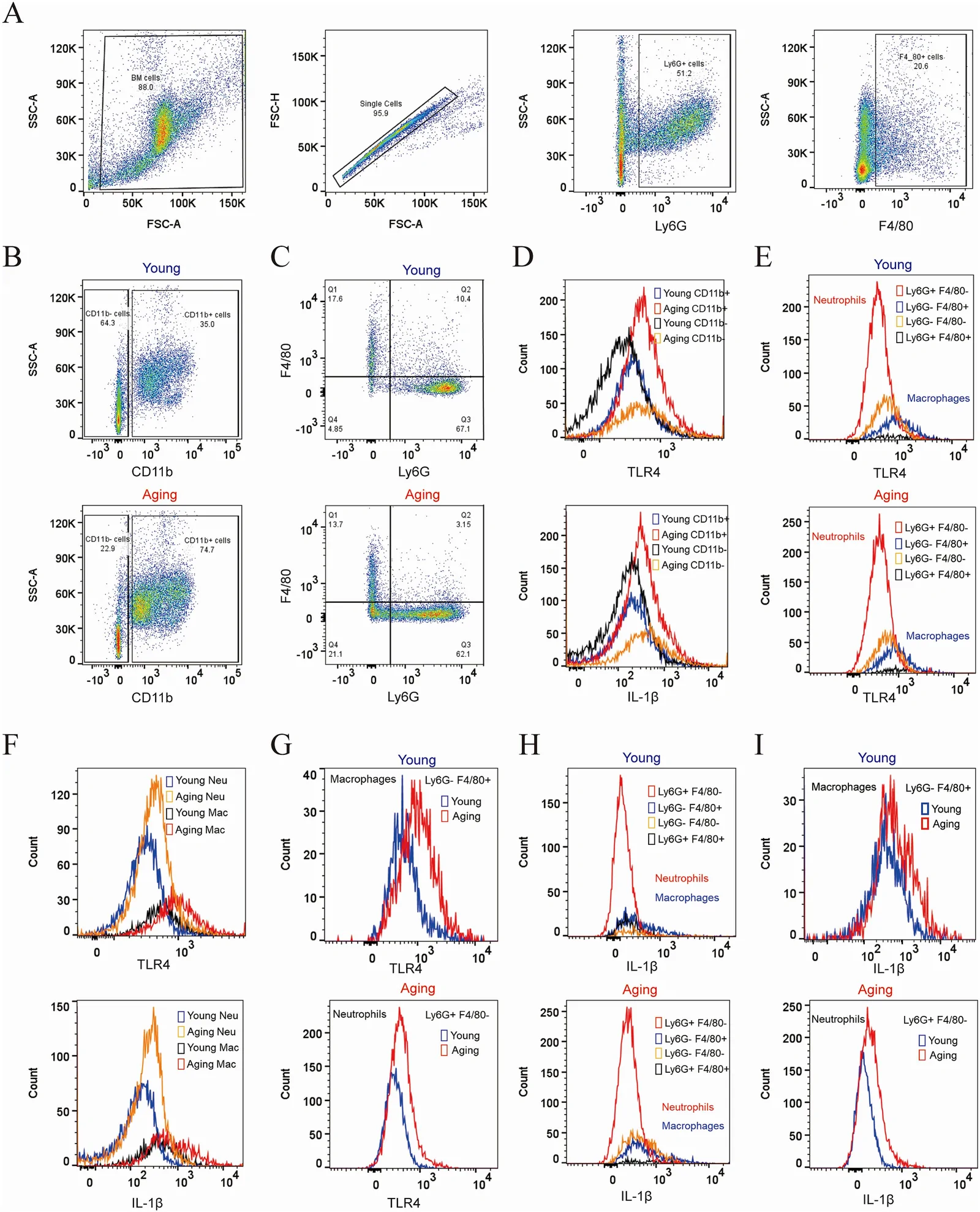
Flow-cytometric analysis of TLR4 and IL-1β expression in bone marrow cells. (A) Gating strategy and single-stain thresholds for Ly6G and F4/80. (B) Bone marrow cells gated by CD11b expression in young (3-month-old) and aged (24-month-old) mice. (C) Subdivision of CD11b^+^ myeloid cells into neutrophil-enriched (CD11b^+^Ly6G^+^F4/80^−^) and macrophage-enriched (CD11b^+^Ly6G^−^F4/80^+^) populations. (D) TLR4 and IL-1β signals in CD11b-negative and CD11b-positive populations and their age-associated differences. (*E*-I) Comparisons of TLR4 and IL-1β signals between neutrophil-enriched and macrophage-enriched populations in young and aged mice.

### 3.2 AT-III suppresses inflammatory responses and osteoclastogenesis in aged bone marrow cultures

CCK-8 analysis showed reduced viability at 200 μM AT-III, whereas concentrations below 100 μM did not produce detectable cytotoxicity during the tested exposure period (Fig. 2A). Accordingly, 25 and 50 μM were used in subsequent assays. AT-III reduced TNF-α and IL-1β concentrations in supernatants from aged BMMs in a dose-associated manner (Fig. 2B). Immunofluorescence further showed lower TLR4 and IL-1β signals after AT-III treatment (Fig. 2C, G, H). Consistent with the cell-culture findings, vertebral immunohistochemistry showed reduced inflammatory staining after 1 month of AT-III treatment in aged mice (Fig. 2D, F).

**Fig. 2:**
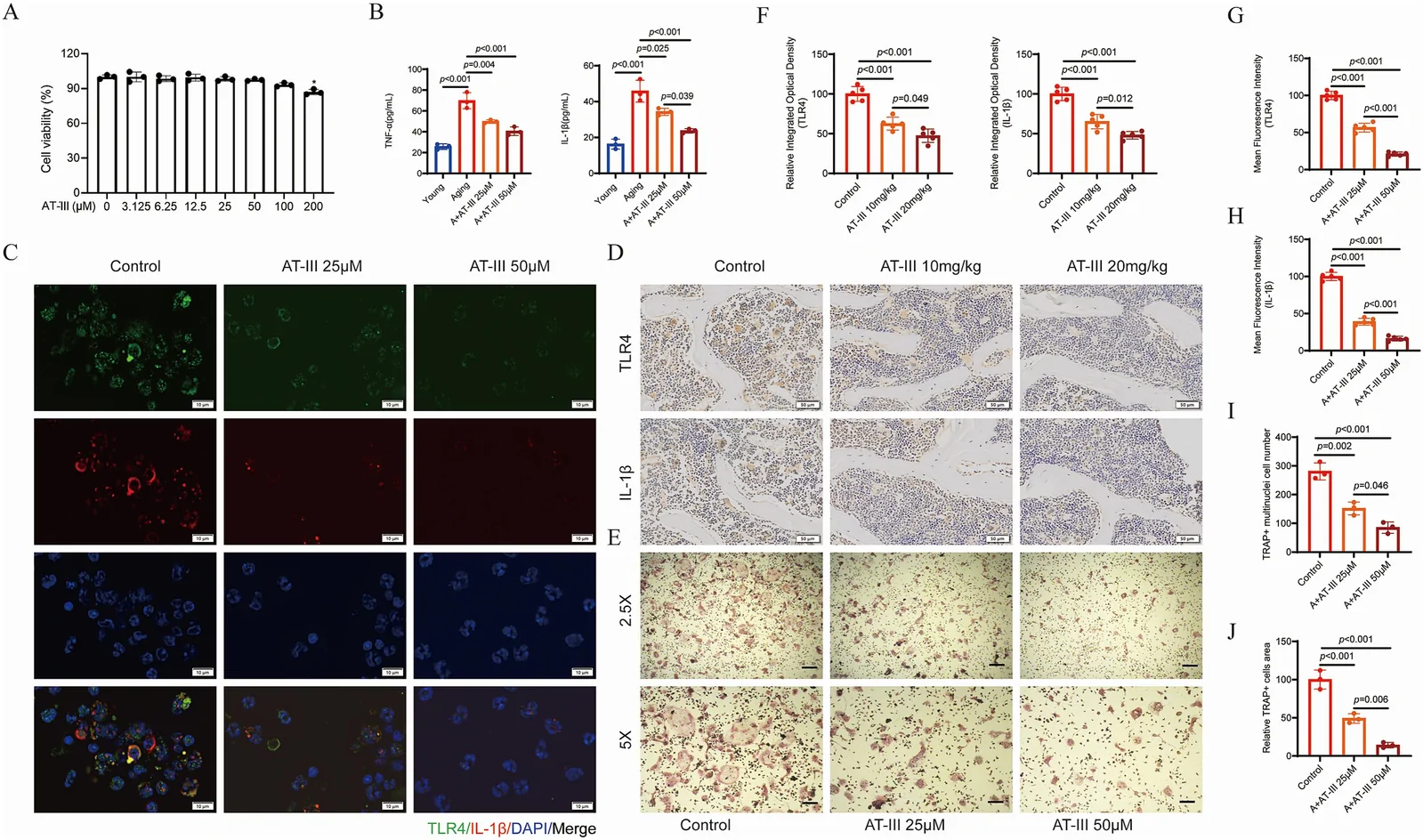
AT-III suppresses inflammatory responses and osteoclastogenesis in aged bone marrow cultures. (A) CCK-8 analysis of BMM viability after exposure to 0–200 μM AT-III. (B) ELISA quantification of TNF-α and IL-1β in supernatants from aged BMMs treated with AT-III (25 or 50 μM). (C) Representative immunofluorescence images of TLR4 (green) and IL-1β (red) after AT-III treatment; scale bar = 10 μm. (D) Representative immunohistochemical images of vertebral sections from vehicle- and AT-III-treated mice; scale bar = 50 μm. (E) Representative TRAP staining after RANKL induction with or without AT-III; scale bars as indicated. (F) Quantification of immunohistochemical staining by integrated optical density. (G, H) Quantification of TLR4 and IL-1β mean fluorescence intensity. (I, J) Numbers and relative areas of TRAP-positive multinucleated cells, averaged from three independent experiments.

Because macrophages are osteoclast precursors, we next tested whether AT-III affected RANKL-induced differentiation. AT-III reduced the number and area of TRAP-positive multinucleated osteoclasts (Fig. 2E, I, J). Together, these results indicate that AT-III suppresses both inflammatory mediator production and osteoclast formation in aged bone marrow-derived cell cultures.

### 3.3 AT-III attenuates vertebral bone deterioration and resorption indices in aged mice

After 1 month of treatment, aged mice receiving AT-III showed improved vertebral microarchitecture compared with vehicle-treated controls. μCT demonstrated dose-associated increases in BV/TV, trabecular number, trabecular thickness, and cortical thickness, together with reduced trabecular separation (Fig. 3). Histological findings were consistent with the μCT results. H&E staining showed better-preserved trabecular architecture, and TRAP staining showed fewer osteoclasts along trabecular surfaces in AT-III-treated mice (Fig. 4A, B). Histomorphometry demonstrated lower Oc.S/BS, Oc.N/BS, and ES/BS and higher Ob.S/BS (Fig. 4C-F). The increase in Ob.S/BS indicates preservation of a bone formation-associated histomorphometric parameter; however, because direct osteoblast differentiation, mineralization, and dynamic bone formation were not assessed in vivo, the present findings alone cannot establish a direct pro-osteogenic effect of AT-III.

**Fig. 3:**
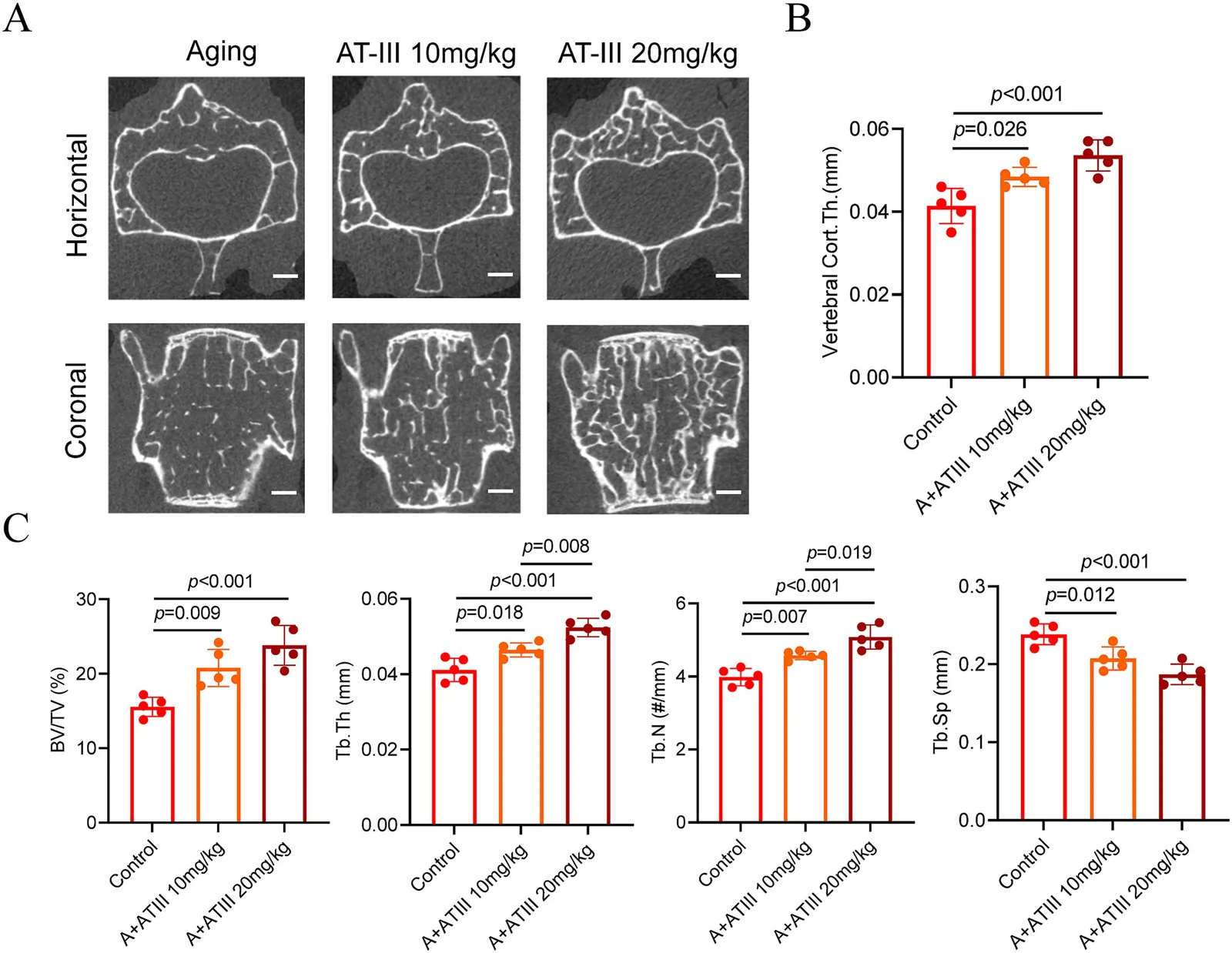
AT-III attenuates vertebral bone deterioration in aged mice. (A) Representative μCT images of L1 vertebrae from vehicle, 10-mg/kg/day AT-III, and 20-mg/kg/day AT-III groups; scale bar = 500 μm. (B) Cortical thickness after 1 month of treatment. (C) BV/TV, Tb.N, Tb.Th, and Tb.Sp in each group.

**Fig. 4:**
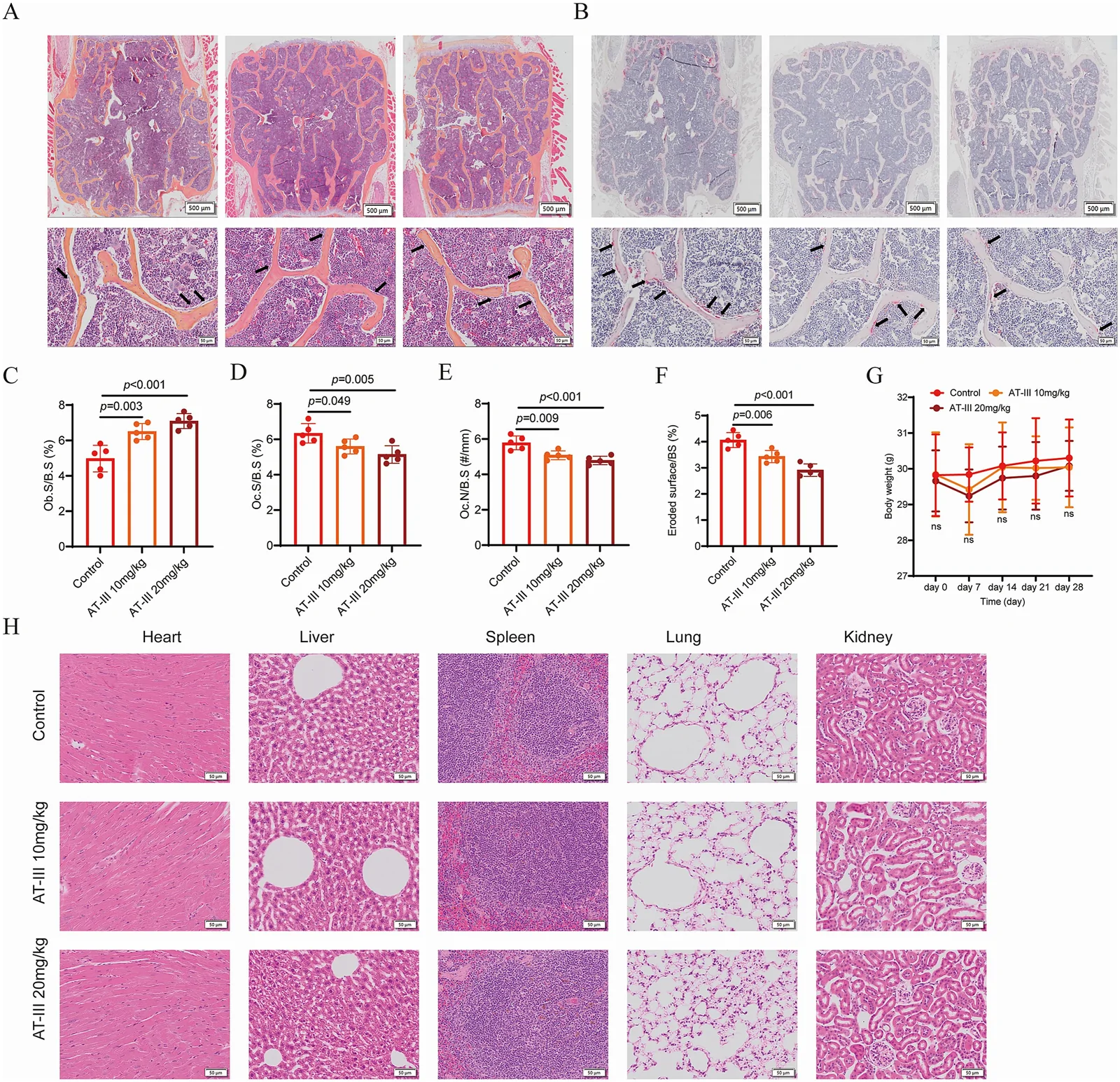
AT-III treatment reduces bone resorption-associated indices without overt short-term toxicity. (A) Representative H&E staining of vertebrae from vehicle- and AT-III-treated aged mice. (B) Representative vertebral TRAP staining. (C—F) Histomorphometric analysis of Ob.S/BS, Oc.S/BS, Oc.N/BS, and ES/BS. (G) Body-weight changes during the 1-month intervention. (H) Representative H&E staining of the heart, liver, spleen, lung, and kidney. Scale bars are indicated in the panels.

Body weight did not differ notably among groups during the intervention, and H&E staining of the heart, liver, spleen, lung, and kidney did not reveal obvious pathological changes under the tested conditions (Fig. 4G, H). These observations do not establish long-term safety but did not identify overt short-term systemic toxicity.

### 3.4 AT-III modestly enhances osteogenic differentiation-associated responses in aged BMSCs

Because AT-III increased osteoblast surface in vivo, we further tested whether it directly affects osteogenic differentiation in vitro. ALP staining showed that 50 μM AT-III significantly increased the ALP-positive area compared with the control group, whereas the effect of 25 μM was modest (Supplementary Fig. 2A, B). Immunofluorescence analysis showed that both 25 and 50 μM AT-III increased COL1 and RUNX2 signals, with a stronger effect at 50 μM (Supplementary Fig. 2C, F). RT-qPCR further showed increased Col1 expression after treatment with 25 and 50 μM AT-III, while Runx2 expression was significantly increased at 50 μM and showed an increasing trend at 25 μM (Supplementary Fig. 2D, E). These findings suggest a modest osteogenic response to AT-III under the tested in vitro conditions.

### 3.5 AT-III downregulates markers of canonical NF-κB pathway activation

Immunoblotting showed that 25 and 50 μM AT-III did not substantially alter total RelA or p50 abundance but reduced their phosphorylated forms in a dose-associated manner (Fig. 5A, B). RT-qPCR showed lower Tlr4, Rela, and Nfkb1 mRNA expression after AT-III treatment (Fig. 5C). These data indicate reduced TLR4/NF-κB-related pathway activity in aged BMMs following AT-III exposure, but they do not by themselves demonstrate direct binding to TLR4 or establish pathway necessity.

**Fig. 5:**
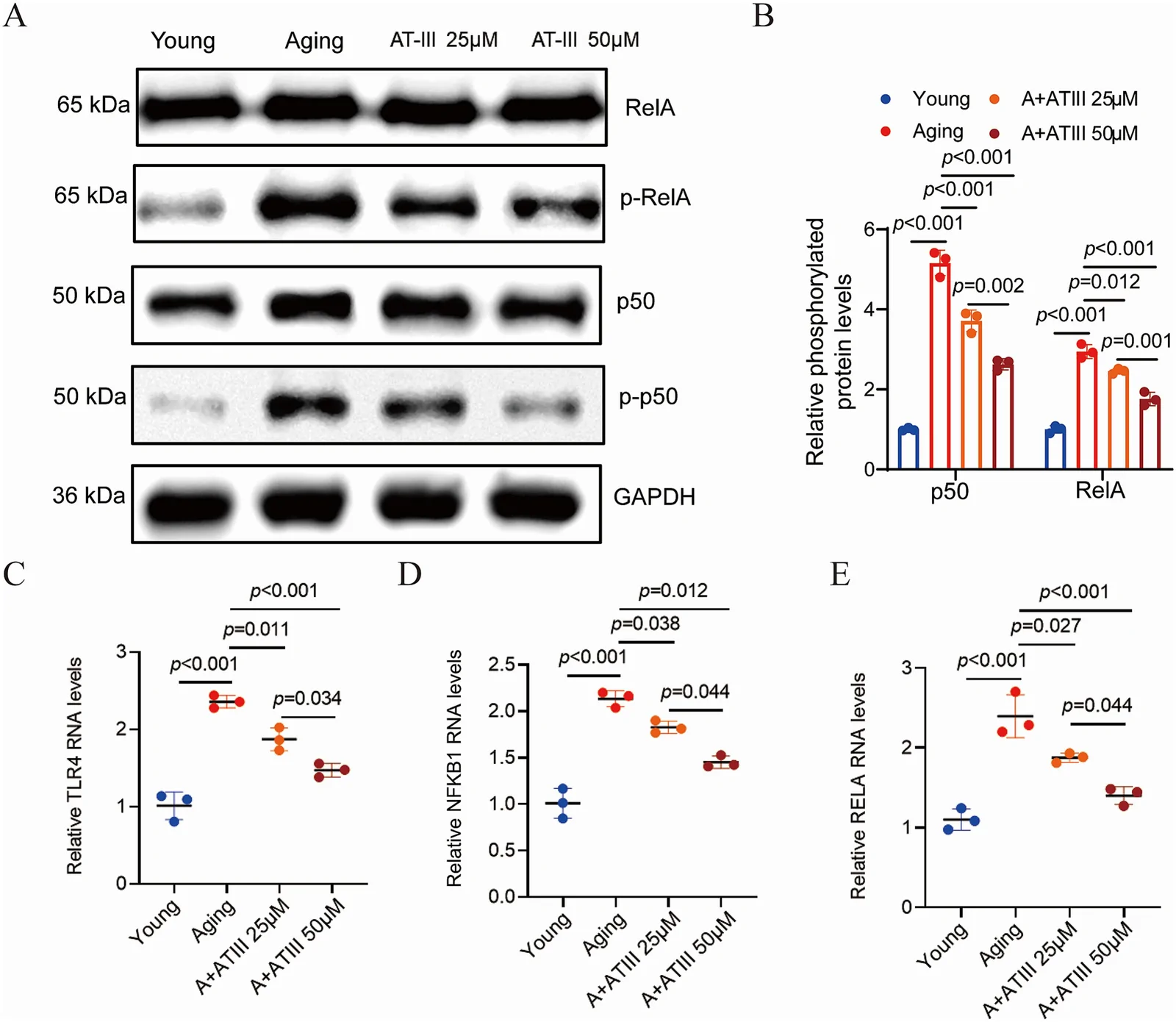
AT-III downregulates markers of canonical NF-κB pathway activation. (A) Representative immunoblots of total and phosphorylated RelA/p65 and p50 in aged BMMs treated with AT-III (25 or 50 μM). (B) Densitometric quantification of p-RelA/RelA and p-p50/p50 ratios. (C) RT-qPCR analysis of Tlr4, Rela, and Nfkb1 expression after AT-III treatment.

### 3.6 Exploratory computational analyses prioritize inflammation-related candidate targets

PharmMapper identified 115 predicted AT-III-associated proteins, and GeneCards yielded 3360 genes associated with age-related osteoporosis. Eighty-one targets overlapped between the two datasets (Supplementary Fig. 1A, B). Network analysis prioritized BCL2, CASP3, EGFR, IL1B, MAPK3, MMP9, NFKB1, SRC, TLR4, and TNF as candidate hubs (Supplementary Fig. 1C). Enrichment analyses highlighted NF-κB, MAPK, PI3K-Akt, and other inflammation-related pathways (Supplementary Fig. 1D-G). Docking generated favorable predicted poses for AT-III with selected candidate proteins, including IL1B, TLR4, and NFKB1 (Supplementary Fig. 1H and Supplementary Table 1). These results informed pathway selection for experimental assessment but were not interpreted as evidence of direct target engagement.

## 4 Discussion

This study provides preclinical evidence that AT-III reduces macrophage-associated inflammation and osteoclastogenesis in the context of age-related bone loss. In naturally aged male mice, AT-III improved vertebral bone microarchitecture and reduced osteoclast-related histomorphometric indices. In aged BMMs, the compound lowered TNF-α and IL-1β production, suppressed RANKL-induced osteoclast formation, and reduced markers of canonical NF-κB activation. These effects were accompanied by an age-associated inflammatory phenotype in macrophage-enriched bone marrow myeloid cells, linking the pharmacological findings to the aged skeletal immune microenvironment.

Previous studies have reported anti-inflammatory effects of AT-III in colitis, asthma, neuroinflammation, and spinal cord injury models (Ren et al., 2021; Zhang et al., 2021a; K. Zhou et al., 2019; X. Zhou et al., 2023; Xue et al., 2022). Our data extend this literature to aged bone marrow macrophages and skeletal remodeling. The present study is distinct from investigations of crude botanical extracts because AT-III is a purified and chemically defined compound. Its effects were evaluated across cellular inflammatory readouts, osteoclast differentiation, and bone structural outcomes, providing a coherent pharmacological phenotype. Nevertheless, the data support a preclinical association rather than established clinical efficacy.

Inflammaging describes the chronic, low-grade inflammatory state that develops with age and contributes to tissue dysfunction (Franceschi, Bonafè, Valensin, et al., 2000, Franceschi, Garagnani, Parini, Giuliani and Santoro, 2018). In aged bone marrow, macrophages may amplify this state while simultaneously providing precursors for osteoclast formation. TLR4-dependent NF-κB signaling is activated by microbial and endogenous danger-associated signals and promotes inflammatory cytokine transcription (Sims et al., 2010; Q. Zhang et al., 2022). In skeletal tissues, excessive TLR4/NF-κB-related activity can disturb osteoimmune homeostasis and favor bone resorption (Muñoz et al., 2020; Y. Zhang et al., 2025). Our observation of increased TLR4 and IL-1β signals in macrophage-enriched cells from aged bone marrow, together with reduced cytokine production and NF-κB phosphorylation after AT-III treatment, is consistent with this framework.

The reduction in osteoclast formation provides a plausible cellular link between the anti-inflammatory effects of AT-III and the improvement in bone microarchitecture. TNF-α and IL-1β can cooperate with RANKL-dependent programs to enhance osteoclast differentiation and activity. AT-III reduced both cytokine secretion and TRAP-positive osteoclast formation, while aged mice showed lower Oc.S/BS, Oc.N/BS, and ES/BS. The present data primarily support anti-inflammatory and anti-osteoclastogenic effects of AT-III. Improvement of the inflammatory bone marrow environment may also indirectly alleviate the suppression of bone formation associated with inflammaging. In addition, our in vitro data showed modest increases in ALP, COL1, and RUNX2, suggesting potential osteogenic activity. However, whether this effect is maintained in the more complex in vivo skeletal environment remains uncertain. Further studies are needed to define the optimal concentration, biological relevance, and underlying mechanisms of this response .

Computational analysis independently prioritized TLR4, IL1B, NFKB1, and inflammation-related pathways. We deliberately treated these results as hypothesis-generating. Molecular docking cannot demonstrate biological binding, and reductions in phosphorylation or transcript abundance do not establish that TLR4/NF-κB is the sole or direct mediator of AT-III action. Rescue experiments using pathway activators, genetic perturbation, target-engagement assays, and pharmacokinetic measurements will be required to define causal molecular mechanisms.

Several limitations should be considered. First, the in vivo study included only a small number of naturally aged male mice, which limits statistical power and requires validation in larger cohorts. Second, skeletal assessment was restricted to the L1 vertebra. Because age-related bone loss is site dependent, findings from a single vertebral site may not represent the entire skeleton; future studies should include additional axial and appendicular sites. Third, only male mice were examined, and further studies in aged females and complementary models, including menopause-associated bone loss, are needed to establish the broader generalizability of these findings. In addition, longer-term safety, fracture resistance, dynamic histomorphometry, and mechanistic validation remain to be investigated.

## 5 Conclusions

AT-III attenuated age-related vertebral bone loss in aged male mice and suppressed inflammatory cytokine production, NF-κB pathway activity markers, and RANKL-induced osteoclastogenesis in aged bone marrow-derived macrophages. These findings support macrophage-associated inflammatory osteoclastogenesis as a pharmacologically responsive component of age-related bone deterioration. Further studies are required to establish direct molecular targets, causal pathway dependence, long-term safety, and efficacy across sexes and additional skeletal aging models.

## CRediT authorship contribution statement

**Cheng Xiang:** Writing – original draft, Visualization, Validation, Resources, Investigation, Formal analysis. **Wenyuan Xiang:** Writing – review & editing, Validation, Resources. **Jannatul Ferdus Shila:** Writing – original draft, Data curation. **Atikul Islam:** Writing – original draft, Data curation. **Yilin Liao:** Writing – original draft, Formal analysis, Data curation. **Zhengxiao Ouyang:** Writing – review & editing, Supervision, Project administration, Funding acquisition, Conceptualization. **Gen Wu:** Writing – review & editing, Project administration, Funding acquisition. **Xinfeng Gao:** Writing – review & editing, Supervision, Project administration, Funding acquisition.

## Funding

This work was supported by the 10.13039/501100001809National Natural Science Foundation of China (grant no. 82371600, Z.O.), the Natural Science Foundation of Wuhan (grant no. 2025020701020270, G.W.), and the Natural Science Foundation of Hubei (grant no. 2026AFC1431, X.F.).

## Declaration of competing interest

The authors declare that they have no known competing financial interests or personal relationships that could have appeared to influence the work reported in this paper.

## Figures and Tables

**Supplementary Fig. 1:**
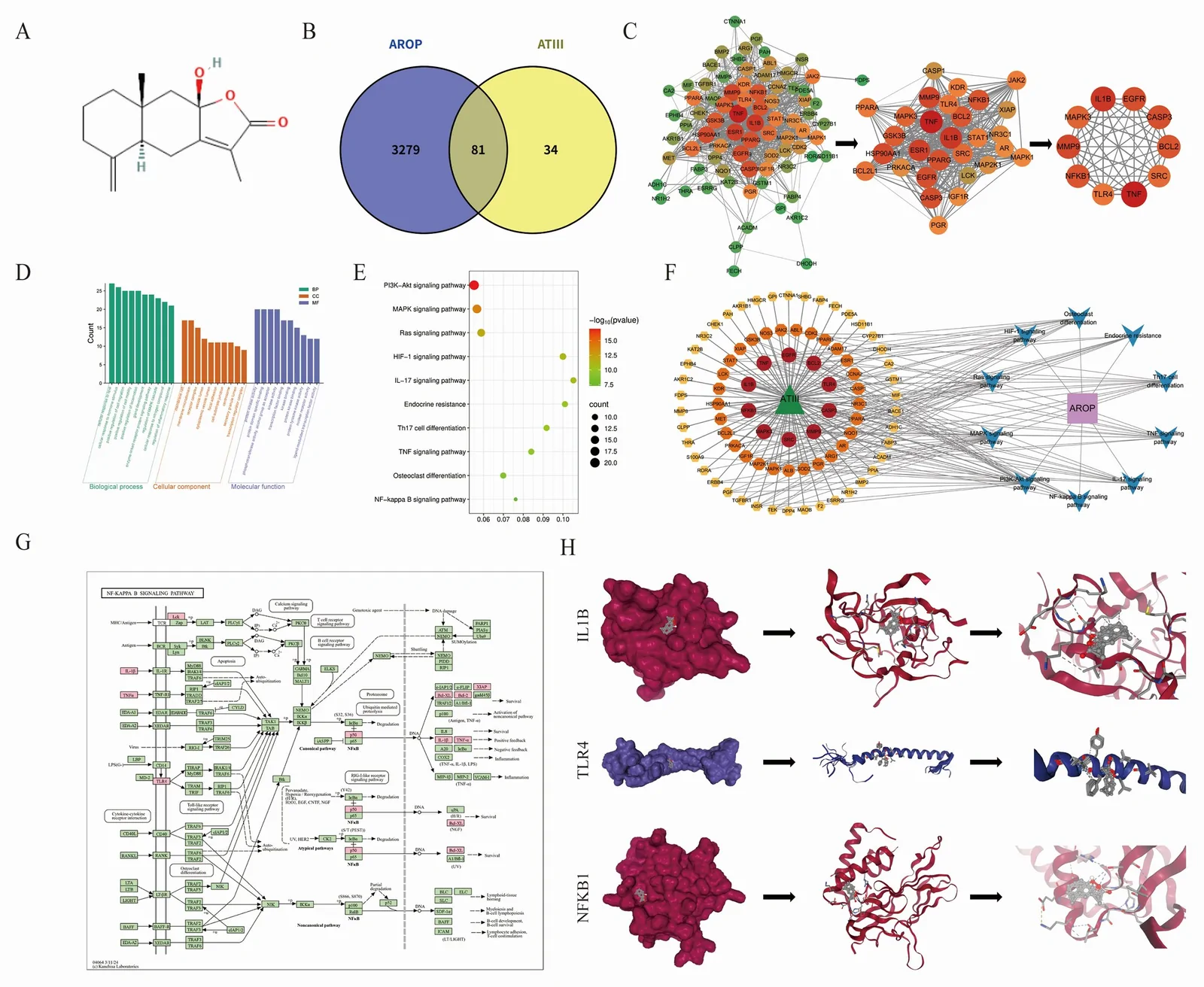
Exploratory network pharmacology and molecular docking analyses. (A) Chemical structure of AT-III. (B) Overlap between predicted AT-III targets and genes associated with age-related osteoporosis. (C) Candidate hub targets identified from the protein-protein interaction network. (D) Representative enriched Gene Ontology terms. (E) Representative enriched KEGG pathways. (F) Compound-target-pathway-disease network. (G) NF-κB pathway map with candidate nodes highlighted. (H) Representative docking poses of AT-III with IL1B, TLR4, and NFKB1.

**Supplementary Fig. 2:**
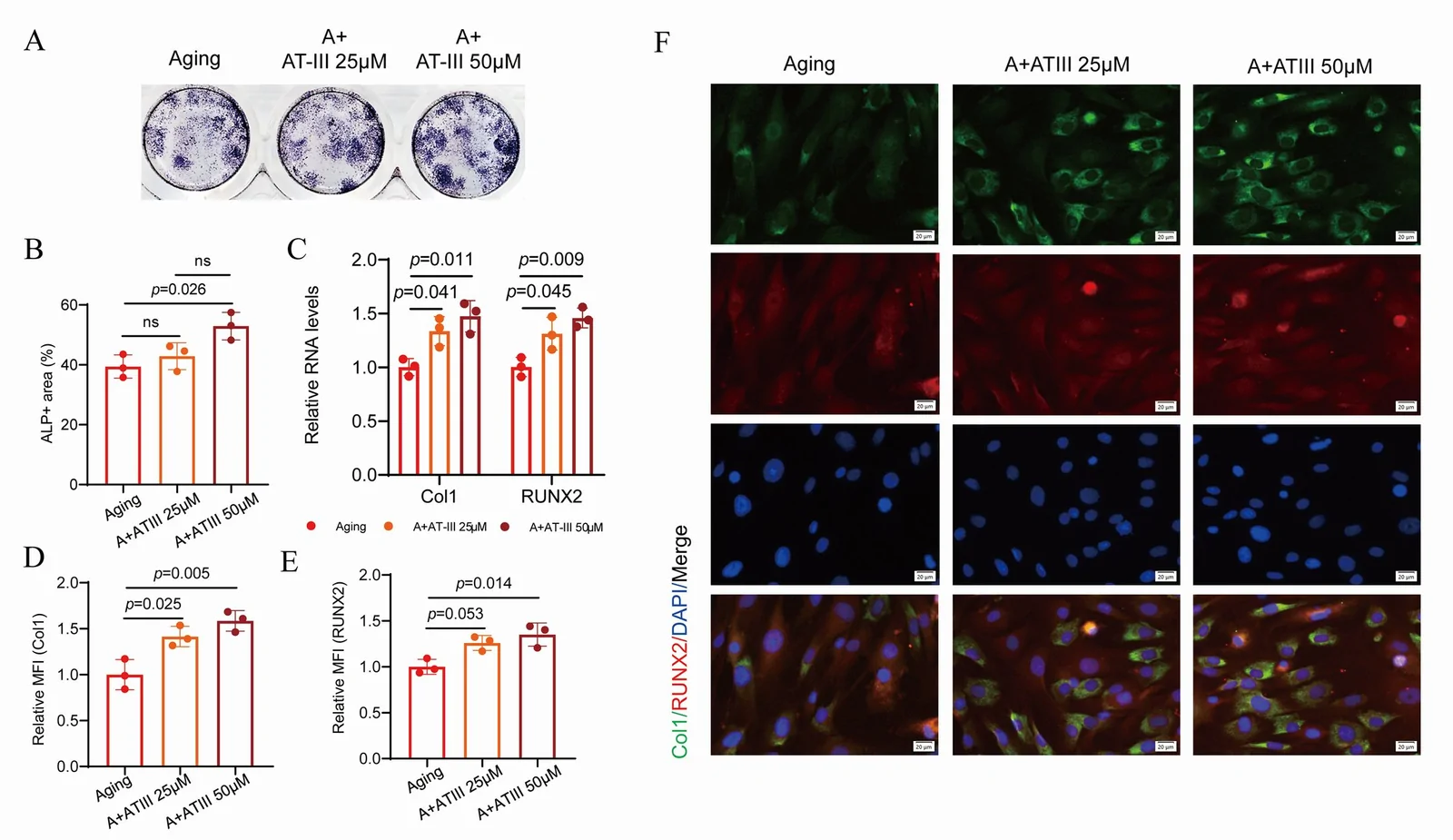
AT-III modestly enhances osteogenic differentiation-associated responses in aged BMSCs in vitro. (A) Representative ALP staining of aged BMSCs treated with vehicle, 25 μM AT-III, or 50 μM AT-III under osteogenic induction conditions. (B) Quantification of ALP-positive area. (C) Quantification of relative COL1 and RUNX2 fluorescence intensity. (D, E) RT-qPCR analysis of Col1 and Runx2 expression. (F) Representative immunofluorescence images of COL1 (green), RUNX2 (red), DAPI (blue), and merged images. Scale bar = 20 μm.

## Data Availability

Data will be made available on request.

## Appendix A Supplementary data

Supplementary Table 1 Predicted molecular docking affinities of AT-III for selected candidate targets.

Supplementary Table 2 Primer sequences used for RT-qPCR.
